# Chitosan-Based Scaffolds Incorporated with Silver Nanoparticles for the Treatment of Infected Wounds

**DOI:** 10.3390/pharmaceutics16030327

**Published:** 2024-02-26

**Authors:** Sibusiso Alven, Blessing Atim Aderibigbe

**Affiliations:** 1 Department of Chemistry, University of Fort Hare, Alice 5700, South Africa; blessingaderibigbe@gmail.com; 2 Department of Chemistry, Nelson Mandela University, Gqeberha 6001, South Africa

**Keywords:** chitosan, silver nanoparticles, infected wounds, hydrogels, nanofibers, sponges

## Abstract

Bacterial infections are major problems in wound care due to their impact on the retarded process of wound healing, leading to chronic wounds. Most of the presently utilized wound dressing products exhibit poor antimicrobial properties. Wound dressings formulated from chitosan have been reported to be effective for treating infected wounds, resulting from the antibacterial properties of chitosan. The antibacterial properties of chitosan-based wound dressings can be further enhanced by incorporating metallic nanoparticles into them, such as silver, zinc, titanium, etc. The incorporation of silver nanoparticles into chitosan-based wound dressings has been widely explored in the design of antimicrobial wound dressings. The incorporation of silver nanoparticles into chitosan-based wound dressings promotes accelerated wound-healing processes due to enhanced antimicrobial activity. The accelerated wound healing by these metal-based nanoparticles is via the regulation of re-epithelialization and inflammation without affecting the viability of normal cells. However, there have been few reports that evaluate these wound dressings in infectious animal models to prove their efficacy. The in vivo toxicity of silver nanoparticles still needs to be addressed, revealing the need for further preclinical and clinical trials. The fabrication of wound dressings incorporated with silver nanoparticles has not been fully explored, especially for wounds requiring immediate treatment. The possible interactions between silver nanoparticles and chitosan scaffolds that result in synergistic effects still need to be understood and studied. This review provides a comprehensive report on the preclinical outcomes of chitosan wound dressing materials loaded with silver nanoparticles for managing infected wounds.

## 1. Introduction

There are several factors, systematic or local, that commonly cause delayed wound healing. Infections remain a considerable challenge in the management of chronic injuries. Patients who suffer chronic injuries, such as burn wounds, suffer from a 75% mortality rate due to infections [1]. Bacterial infections hinder innate inflammatory pathways and can result in the microbes developing resistance against conventional antimicrobial therapeutics [2,3]. Infections are an important cause of morbidity in chronic wound patients with several consequences, such as delayed healing, hospitalization, and amputation. Conditions such as osteomyelitis, cellulitis, and abscesses need instant medical attention [4]. The severity of the burden results from the increased incidence of infections related to multidrug-resistant (MDR) bacteria. The World Health Organization (WHO) announced that more than two million infections are caused by MDR bacteria, with direct and indirect costs surpassing USD 55 billion [5].

Wound dressings play a vital role in wound repair by preventing microbial infections, invasion of foreign substances, and further skin tears. They also contribute to the restoration of skin layers. Preclinical studies have shown that wound dressings formulated from natural polymers display most of the properties of ideal wound dressings. Natural polymers are obtained from natural sources, such as animals, plants, and microorganisms. These polymers mimic the original extracellular matrix (ECM). They undergo biochemical degradation that modifies their physicochemical features and interaction with surrounding tissues in a physiological environment [6,7]. Natural polymers include chitosan, alginate, cellulose, gelatin, dextran, gelatin, hyaluronic acid, etc. [7].

Chitosan-based materials have interesting properties that make them suitable for several biomedical applications, including wound healing, etc. [8]. These materials exhibit controlled drug release mechanisms at selected temperatures and pH values, revealing a dual-responsive drug release profile [9]. They display good biocompatibility with excellent blood coagulation capability, making them appropriate for wound dressing applications [9,10,11]. Chitosan possesses good biocompatibility and biodegradability [12,13,14]. Chitosan-based wound dressings have been loaded with metallic nanoparticles for the treatment of wounds. Silver nanoparticles (AgNPs) are potential antimicrobial nanomaterials widely explored for the treatment of bacterial infections [15]. Factors that expose wounds to infections are contaminated environments, prolonged hospitalization, poor hygiene, poor management of wound exudates, etc. [16]. Treating microbial infections is a good approach to accelerating the rate of wound healing because infections delay the wound-healing process, resulting in sepsis and amputation [17]. In clinical practice, a thorough assessment of the wound, the use of appropriate wound dressings that maintain a moist environment for clean wounds, and debriding wound dressings are effective approaches for controlling infections [18]. The design of chitosan-based scaffolds incorporated with AgNPs has the potential to further enhance their antimicrobial activity and accelerate the rate of wound healing due to possible synergistic effects. This review reports on chitosan-based scaffolds loaded with Ag nanoparticles, with a promising potential to treat infected wounds.

## 2. The Mechanism of the Wound-Healing Process

**Figure 1:**
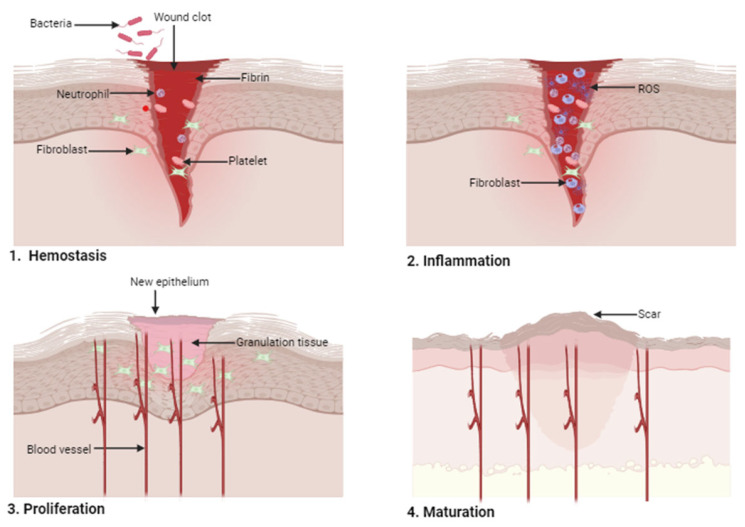
Sequential phases of wound healing.

Wound dressings are primarily used to accelerate the process of wound healing. The wound-healing process is a multifaceted mechanism involving the interaction of cells and growth factors. Four phases of wound healing overlap: hemostasis, inflammation, proliferation, and maturation phases [19]. Figure 1 is a schematic presentation of the four phases of wound healing. The hemostasis phase occurs instantly to terminate the bleeding, which depends on the coagulation cascade, platelet activity, fibrin clot formation, and blood vessel constriction [20]. The clot formation protects the wound from the invasion of exogenous agents, reduces fluid and blood loss, and offers a provisional matrix that initiates the healing process. Neutrophils and macrophages are recruited, and they secrete signaling factors that manage the next phases of the wound-healing process [21].

The second phase of the wound-healing process is inflammation, which normally overlaps with the first phase. At this phase, some immune cells contract the wound area and act as an immune barrier against microorganisms. The leukocytes (specifically neutrophils) also invade the wound site to eradicate debris and bacteria, and cytokines are released by neutrophils as an inflammatory response [22]. Simultaneously, monocytes move into the wound site and differentiate into macrophages, recruiting more monocytes. Monocytes, neutrophils, and macrophages play a vital role at this stage by secreting proinflammatory cytokines and growth factors that activate epithelial cells and fibroblasts that are required in the next wound-healing phase [23]. There are microorganisms (i.e., bacteria) that are commonly found on the skin. These microorganisms have access to the wound after skin injury, leading to infections. These infections play a major role in delaying the inflammation phase during the wound-healing process. These bacteria produce virulence factors and endotoxins and promote the expression of pro-inflammatory cytokines, a major cause of chronic inflammation. In addition, prolonged inflammation leads to disordered metabolism, such as high matrix metalloproteinases, and delays the normal healing process [24]. The transition of macrophages to induce endothelial cells, fibroblast, and keratinocytes, a process useful in stimulating tissue regeneration, is delayed in infected wounds because of the increased burden on the immune cells that disrupt attacking bacteria, resulting in a challenge of planktonic bacteria producing biofilms, potentially causing wound chronicity. In patients, the prolonged inflammatory reactions caused by infections can lead to foul smell, pain, and prolonged length of hospitalization [25,26]. Chronic wounds, such as diabetic foot ulcers, are characterized by a prolonged inflammation phase, resulting from high amounts of neutrophils, macrophages, and proinflammatory cytokines in the wound bed, causing an impaired wound-healing process [27]. Furthermore, the continuous inflammatory response also increases ROS and delays the wound-healing process. In acute wounds, the inflammatory stage is not prolonged because the M2 macrophages replace M1 macrophages when compared to predominate M1 macrophages in the wound microenvironment of diabetic foot ulcers [27].

The proliferation phase promotes the reduction of damaged skin. This stage is characterized by angiogenesis, collagen deposition, granulation tissue development, and epidermal regeneration [28]. The fibroblast moves to the wound site, promoting ECM proteins (proteoglycan fibronectin and hyaluronan) to substitute fibrin clots. Collagen development provides strength to the tissue. The fibroblasts are differentiated into myofibroblasts, reducing the proliferation and increasing the development of collagen, contracting the injury, and decreasing the size of the wound [29]. The last phase of the wound-healing process is the maturation phase (remodeling phase), whereby the ECM is slowly altered into a complete scar. In this phase, the collagen is restructured and the collagen type III developed in the ECM is substituted by collagen I (this alteration occurs with the reduction of the lesion), with the skin obtaining approximately 80% of its original tensile strength [30,31].

## 3. Classification of Wound Dressings

Wound dressings are generally categorized into five groups: skin substitutes, traditional dressings, interactive dressings, dermal grafts, and bioactive wound dressings [32]. Traditional wound dressings (passive dressings) protect the injury from contamination or foreign substances, cushion the injury, absorb exudates, and stop bleeding. Gauze, wool dressing, bandages, plaster, and gauze are examples of traditional wound dressings [33]. The limitations of traditional dressings are leakage of wound exudate that exposes the wound to bacterial invasion and the pain they cause upon removal [34]. Table 1 is a summary of the classes of wound dressings [33,34,35,36,37,38,39].

Skin substitutes are tissue-engineered materials that are derived from cell-seeded scaffolds and are effective in the regenerative healing of the skin. Nevertheless, their use can result in the transmission of diseases and wound infections and they are expensive, can be rejected by the host, and possess inadequate shelf life. Some examples of skin substitutes include TransCyte, OrCel, and Apligraf [35]. Dermal grafts are biomaterials that are useful for plastic surgery and dermatology; their examples are acellular xenografts, allografts, and autografts [36]. These biomaterials are used to treat traumatic lesions, congenital skin deficiencies, post-oncologic resection, hair restoration, burn reconstruction, scars, and vitiligo. The drawback of dermal grafts is their inability to treat complex wounds involving deep spaces and exposed bones [36].

Interactive wound dressings accelerate the wound-healing process by providing a moist environment, improving re-epithelialization and granulation, and offering a good water transmission rate. Examples of interactive dressings include sprays, films, foams, and gels [37]. These wound dressing materials can be incorporated with drugs to form bioactive wound dressings. Bioactive dressings such as hydrocolloids, sponges, hydrogels composites, wafers, foams, nanofibers, membranes, and films are biodegradable, biocompatible, and act as drug delivery systems for various bioactive molecules, such as growth factors (GFs), nanoparticles, vitamins, and essential oils, to enhance the wound-healing process [38,39].

Most of these wound dressing types have been extensively used to prevent and treat infected wounds. Traditional and interactive wound dressings possess the potential to prevent wound infections by protecting lesion beds from the invasion of bacteria. Bioactive dressings can be used to treat infected wounds because they are loaded with various bioactive agents (e.g., antibiotics, metallic nanoparticles, and plant extracts) that exhibit antimicrobial activity [40]. Skin substitutes and dermal grafts are not suitable for the treatment and prevention of infections on wound beds. They are extensively used in the treatment of wound types, including traumatic lesions, post-oncologic resection, burns, congenital skin deficiencies, etc. [36]. The current clinical need that should be focused on is the development of advanced wound dressings that maintain a moist wound environment, have a good gaseous exchange, are easy to remove after wound recovery, accelerate the wound-healing process, promote good absorption of exudates, and exhibit antibacterial activity. The aforementioned features are important for accelerated wound healing rates, including infected wounds [38].

Wound debridement also plays a vital role in the prevention and treatment of infected wounds. These techniques include mechanical debridement (i.e., the application of both dry gauze and wet-to-dry dressings to promote the elimination of infected tissue), biological debridement (i.e., the use of enzymatic, autolytic, and maggot and honey therapies), surgical debridement (the direct excision of all necrotic and infected tissue within an injury utilizing a combination of mayo scissors, scalpel blades, power burrs, sagittal saws, curettes, and hydrosurgical instruments), and enzymatic debridement (i.e., the application of chemical substances to break down devitalized tissue) [41,42,43].

## 4. Properties of Chitosan

**Figure 2:**
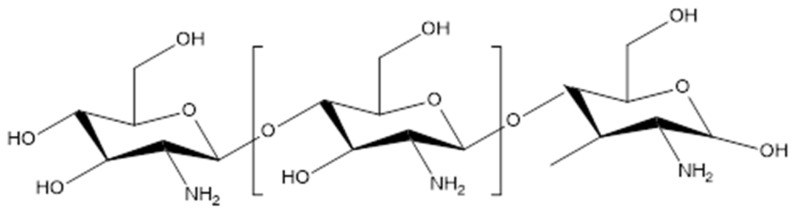
Molecular structure of chitosan.

Chitosan (Figure 2) is a linear amino biopolymer prepared by alkaline chitin *N*-deacetylation extracted from the exoskeleton of crustaceans, such as shrimps, lobsters, and crabs [44]. The chitosan derivatives can be simply synthesized by chemically modifying the amino- and hydroxyl-groups available in the polysaccharide. Chitosan derivatives include *N*-succinyl-, *N*-carboxymethyl-, *N*-carboxybutyl-, *N*-acyl-, 5-methylpyrrolidinone-, *N*-carboxyethyl-, *O*-carboxymethyl-, *O*-succinyl-, and *N*,*N*-dicarboxymethyl-chitosan derivatives [45]. Chitosan and its derivatives have been extensively explored for several biomedical applications due to their functionalities; biodegradability; biocompatibility; non-toxic nature; and antioxidant, antimicrobial, and bio-adhesive properties. They are also considered an important biomaterial for developing wound dressings due to their wound-healing properties [46].

Chitosan is used to develop various scaffolds, such as nanofibers, sponges, films, hydrogels, membranes, wafers, etc. Like other natural polymers, chitosan scaffolds suffer from weak mechanical performance and can undergo distortion via externally applied stress. This drawback can be solved by combining it with other appropriate materials, especially synthetic polymers, to enhance its mechanical features for the formulation of wound dressing materials [47]. Chitosan is soluble in an aqueous acidic medium at a 50% degree of deacetylation or higher, depending on the polymer origin; as a result, its amino groups possess a pKa value of 6.3. Chitosan solubility is high in 0.1 M or 1% acetic acid [48].

Chitosan possesses antibacterial features that have attracted important attention from biomedical researchers in various biomedical applications, including wound healing, etc. The antimicrobial effect of chitosan is attributed to its cationic nature, making it an ideal biomaterial for the development of wound dressings. The ability of chitosan to hinder the growth of a broad diversity of bacteria, yeasts, viruses, and fungi makes its application useful in a wide variety of antimicrobial agents in various forms (e.g., solutions, composites, gels, and films) [49]. The antimicrobial effects are governed by fundamental factors, including the degree of chitosan polymerization, the type of chitosan, the molecular weight, the environmental pH, etc. [50]. Chitosan also plays an essential role in wound healing as a haemostatic biomaterial, specifically for bleeding wounds. The coagulation activity of chitosan is due to its ability to offer many active sites for erythrocytes and stimulate platelets, which results in the development of a fibrin clot to terminate hemorrhage [51]. The gelation property of chitosan scaffolds is one significant aspect in biomedical fields that mainly depends on the pH of the environment, among other conditions. The short gelation time of hydrogels is very crucial, specifically for injectable hydrogels, to prevent the washout of cells and extrusion in the injection site. Sodium hydroxide (NaOH) can be used as a strong base to induce physical crosslinking between polymeric chains of chitosan via hydrogen bonding, leading to instant gelation. Phosphate buffer solutions are normally used to adjust the pH of hydrogel to around 7.4, an ideal physiological pH where instant gelation of chitosan takes place [52,53]. Chitosan scaffolds can be incorporated with metallic nanoparticles to further enhance their antimicrobial efficacy.

## 5. Chitosan-Based Scaffolds Incorporated with Silver Nanoparticles

Wound dressing scaffolds have been loaded with AgNPs, specifically for the treatment of infected wounds. Due to their multi-site action, AgNPs display broad-spectrum antibacterial effects against different bacteria strains. The antibacterial properties of AgNPs have resulted in their escalated demand in recent years for wound healing, drug carriers, etc. [54]. The mode of action of AgNPs in various biological systems results from the increased surface area and nanometric size and their capability to disrupt the membrane, cross the microbe body, and cause intracellular death [55]. The entire breakdown of the cells and the elimination of lipopolysaccharide happens via membrane protrusions binding to nanoparticles, which enter the cell by electrostatic attraction. Metal ion release, non-oxidative mechanisms, and oxidative stress induction are also recognized modes of action for defining the antimicrobial effects of AgNPs [56]. Nevertheless, the toxic effects of AgNPs in wound healing applications should be studied. Various mechanisms of AgNPs cytotoxicity include (i) their ability to adhere to the bacterial cell membrane, causing physical disruption and impairment of bacterial cell membrane; (ii) cellular internalization that can result in the malfunction of some cell organelles (ribosomes, mitochondria, and vacuoles) and biomolecules (DNA, enzymes, and proteins); (iii) generation of free radical and ROS that can also destroy the activity of intracellular organelles and biomolecules; and (iv) the modulation of intracellular pathways [57,58]. Recently, it has been reported that the mechanism of action of Ag-containing biomaterials involves the peroxidase activity of O_2_ nanobubbles, resulting in the formation of more ROS. The generation of ROS induced by Ag-containing materials in the bacteria, along with the interruption of the electron transport chain, damages the bacterial membrane integrity and eventually destroys the bacterial cell [59]. The controlled release of Ag nanoparticles from biomaterials is another important feature that can determine the degree/extent of the antimicrobial efficacy as well as the biocompatibility. Several factors influence the controlled release of Ag, including the molecular mass of the used biomaterials, the pH of the suspension medium, the initial pH of the biomaterial, the presence of a crosslinking agent, the loaded amount of Ag into the biomaterials, etc. [60].

### 5.1. Sponges

**Figure 3:**
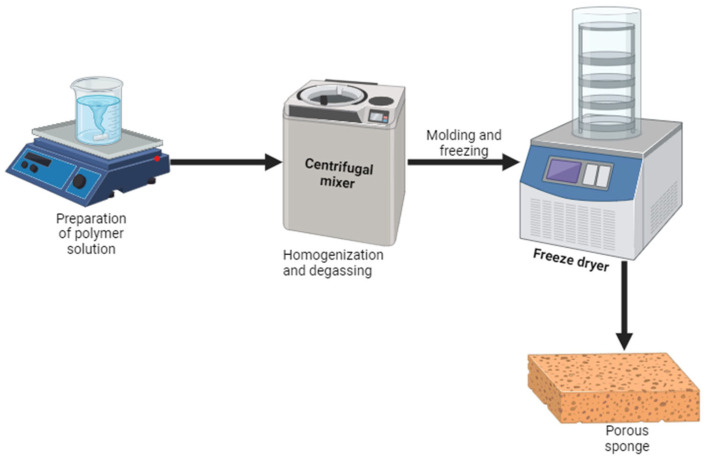
Schematic diagram showing the preparation of porous sponges.

Sponges are wound dressings with a porous structure. The most common method used to prepare sponges is the freeze-drying method, also known as lyophilization [61]. A schematic diagram showing the preparation of the porous sponges is shown in Figure 3. Their high porosity permits the absorption of massive volumes of biological fluids, including exudates, making them appropriate for highly exuding wounds [62]. However, sponges alone cannot maintain a moist environment because of their low water content. They are ineffective in the management of dry necrotic injuries [63]. Their mechanical properties can be enhanced by increasing the density of crosslinking to form a stable three-dimensional (3D) network [64]. The preclinical studies of chitosan-based sponges loaded with AgNPs demonstrated promising therapeutic outcomes for the management of infected injuries. Ye et al. reported chitosan/gelatin sponges incorporated with AgNPs with a non-toxic effect on mouse fibroblasts (L929 cells), with more than 80% cell viability after 24 h incubation [65]. The antibacterial analysis of AgNP-loaded nanofibers showed good antimicrobial efficacy against *Staphylococcus aureus* (*S. aureus*) and *Escherichia coli* (*E. coli*), which was improved as the content of Ag nanoparticles increased [65].

Jiang et al. fabricated chitosan/alginate composite sponges incorporated with a combination of curcumin and AgNPs to treat infected wounds [66]. The AgNPs were synthesized using sericin. The porosity of the composite was 60.4 ± 5.2%, enhancing the increased content of AgNPs and curcumin. The porosity of the composite sponges provides a suitable circulation of oxygen and nutrients during wound healing. The in vitro antimicrobial studies utilizing an agar plate procedure showed that the dual drug-loaded composite sponges possess superior antibacterial activity against *P. aeruginosa* and *S. aureus* compared to single drug-incorporated and plain sponges. These antimicrobial results reveal a synergistic antibacterial effect of chitosan when loaded with bioactive agents, a promising feature for treating infected wounds [66]. The chitosan sponges loaded with iturin-synthesized AgNPs were reported by Zhou et al. to exhibit efficient inhibition against *S. aureus* and *P. aeruginosa.* The sponges also stimulated the process of wound healing in vivo [67].

Liu et al. formulated chitosan/silk fibroin hybrid sponges incorporated with AgNPs. The in vitro drug release evaluation of the hybrid sponges showed a slow release of AgNPs from the sponges, revealing their capacity to protect wounds from bacterial invasion. The antimicrobial analysis of the AgNP-loaded sponges showed superior antimicrobial efficacy against *S. aureus*, *E. coli*, *C. albicans*, and *P. aeruginosa*. The in vivo experiments showed that the hybrid sponges containing AgNPs promoted a wound closure of 99.38% on the 14th postoperative day, while the plain sponges induced only an 89.22% wound closure [68]. Lu et al. reported chitosan-based sponges incorporated with a combination of Ag and ZnO nanoparticles for wound dressing applications. The in vitro antibacterial results of the dual nanoparticle-loaded sponges using an inhibition procedure showed superior antibacterial effects against *S. aureus*, *P. aeruginosa*, *E. coli*, and *Methicillin-resistant Staphylococcus aureus* (MRSA) than the plain sponges. The in vivo wound closure analysis showed that the dual nanoparticle-loaded sponges exhibited healing on the third-day post-surgery than the ZnO ointment gauze and plain chitosan [69]. Huang et al. formulated chitosan-based sponges embedded with AgNPs for antibacterial wound dressing. The in vitro studies showed more than 99% antimicrobial efficacy against *S. aureus* and *E. coli*, with excellent biocompatibility and non-toxicity when incubated with MC3T3 cells [70].

Microfibrillated, cellulose-reinforced chitosan-based sponges loaded with a combination of AgNPs and recombinant humanized collagen type III were reported by Long et al. These sponges demonstrated effective antibacterial effects and significantly stimulated the in vitro cell migration and proliferation of L929 cells [71]. Lu et al. formulated chitosan-*L*-glutamic acid/hyaluronic acid sponges incorporated with AgNPs. The sponges displayed interconnected porous morphology, indicating their capacity to promote gaseous exchange and diffusion of nutrients and skin cells during wound healing. The antibacterial analysis showed that the composite sponges loaded with AgNPs effectively inhibited the bacterial growth of *E. coli* and *S. aureus* bacterial growth. The in vivo studies employing full-thickness lesions in rabbits revealed rapid wound contraction when treated with AgNP-loaded sponges (i.e., approximately 69% wound closure on day 3) than those treated with pristine sponges (with only 47% wound contraction) [72].

Wu et al. developed AgNP-incorporated chitosan sponges that displayed a high porosity of approximately 99.42% and excellent antimicrobial activity against *S. aureus*, *P. aeruginosa*, and *E. coli* [73]. Ding et al. formulated chitosan/*Bletilla striata* polysaccharide hybrid sponges incorporated with AgNPs. The in vitro antimicrobial studies showed that the hybrid sponges loaded with Ag nanoparticles were effective against *E. coli*, *P. aeruginosa*, and *S. aureus*, while the plain sponges displayed no significant antibacterial effects. The in vivo studies utilizing full-thickness cutaneous wound healing in Kunming mice revealed that the AgNP-loaded sponges stimulated a faster wound healing rate than the control, a medical gauze [74]. Chabala et al. prepared lyophilized chitosan/alginate sponges enriched with a combination of AgNPs and Aloe vera for wound healing applications. The in vitro antimicrobial results of the dual drug-loaded composite sponges showed greater bacterial inhibitory capacity than gentamicin [75].

The in vitro studies of chitosan-based sponges loaded with Ag nanoparticles showed more than 70% cell viability of various skin cells, revealing good cytocompatibility/biocompatibility and non-toxicity. The antimicrobial analyses revealed promising antibacterial efficacy against different strains (i.e., *S. aureus* and *E. coli*) that are common in clinical cases, suggesting that Ag nanoparticle-loaded chitosan sponges are potential wound dressings that can be used for the prevention and treatment of infected wounds. Chitosan-based sponges also displayed high porosity, an important feature associated with improved cell migration/proliferation and gaseous exchange during the wound-healing process. Although the combination of AgNPs and other bioactive agents in the chitosan sponges significantly enhanced the antimicrobial and wound healing activity, their mode of action is not fully understood.

### 5.2. Nanofibers

**Figure 4:**
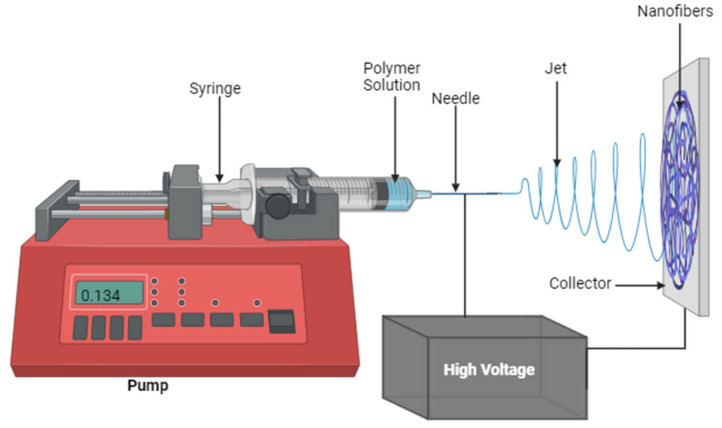
Schematic diagram showing the electrospinning technique.

Chitosan-based nanofibrous wound dressings have been widely fabricated by electrospinning (Figure 4). Electrospinning is an efficient method for preparing polymeric fibers with a nano-scale range. Some fascinating advantages of electrospinning are the easy loading of bioactive agents into the nanofibers and its cost-effectiveness, and it does not require heat, a crucial advantage for sensitive bioactive agents [56,76]. The electrospun nanofibers possess unique features, including a large surface area-to-volume ratio, high porosity, small pore size, and the capacity to be loaded with various bioactive compounds. These features of electrospun nanofibrous materials are being explored in wound healing, drug delivery, and tissue regeneration [77,78]. Currently, there are several reports exploring chitosan-based nanofibers incorporated with AgNPs for the treatment of infected wounds.

Fereydouni et al. prepared chitosan/polyethylene oxide (PEO) nanofibers loaded with AgNPs. The mechanical properties of the chitosan/PEO nanofibers showed tensile strength ranging between 7.14 ± 0.4 and 10.48 ± 1.53 MPa, similar to human skin, which is in the range of 5–30 MPa. The AgNP-loaded nanofibers showed significant antibacterial efficacy against *P. aeruginosa* and *S. aureus*, revealing their potential to manage infected injuries, together with excellent mechanical properties [79]. Foroushani et al. reported chitosan/silk fibroin nanofibers loaded with a combination of AgNPs and curcumin that demonstrated strong inhibitory effects against *E. coli* and *S. aureus* [80].

Ganesh et al. developed chitosan/polyvinyl alcohol (PVA) loaded with a combination of AgNPs and sulfanilamide, using the electrospinning method. The drug release kinetics at the physiological environment of pH 7.4 and 37 °C showed an initial rapid drug release of the loaded bioactive agents from the nanofibers, followed by a sustained release mechanism, indicating their capability to destroy bacteria and further protect the wound from bacterial invasion [81]. Lee et al. formulated chitosan-based nanofibers incorporated with Ag nanoparticles that demonstrated effective antibacterial effects against MRSA and *P. aeruginosa*, indicating their potential application as antibacterial wound dressings [82].

The AgNP-loaded chitosan/PEO hybrid nanofibers reported by Aljohani et al. showed excellent biocompatibility towards keratinocyte cells and human skin fibroblast lines, with more than 93.5% cell viability and superior antibacterial efficacy against *S. aureus* and *E. coli*, suggesting a superior antimicrobial effect to eliminate wound infections with reduced adverse effects [83]. Junior et al. fabricated chitosan/poly (lactic acid) (PLA) nanofibrous materials loaded with a combination of AgNPs and chondroitin-4-sulfate for infected wound care. The nanofibers showed smooth fibers with a diameter of 340 ± 18 nm and a porosity of 89 ± 3.08%, suggesting their capability to allow the circulation of gases and cells during wound healing. The dual drug-loaded nanofibers exhibited good antimicrobial efficacy against *S. aureus* and *E. coli*, with excellent cytocompatibility towards L-929 fibroblast cells [84].

Hussein et al. reported chitosan/PVA/PCL hybrid nanofibers co-incorporated with AgNPs and phenytoin for wound treatment. The in vitro drug release profile was a controlled release of phenytoin from the dual drug-loaded nanofibers. The dual drug-loaded hybrid nanofibers showed good antibacterial activity against *S. aureus* and *E. coli*, but the antimicrobial activity was slightly higher against *S. aureus* than against *E. coli* [85]. Kohsari et al. synthesized electrospun chitosan/PEO nanofibrous mats incorporated with green synthesized AgNPs using *F. vulgaris* herbal extract. The nanofibers demonstrated excellent antibacterial effects against *E. coli* and *S. aureus* [86]. The chitosan/PEO hybrid nanofiber co-loaded with Ag and ZnO nanoparticles was reported by Bagheri et al. They exhibited superior antimicrobial efficacy against *S. aureus*, *P. aeruginosa*, and *E. coli*, with excellent cytocompatibility on the fibroblast cells [87]. Wang et al. prepared chitosan/PEO nanofiber mats that promoted a sustained drug release of AgNPs for three days, with excellent antimicrobial efficacy against *S. aureus* and *E. coli* [88].

Abdelgawad et al. prepared chitosan/PVA hybrid nanofiber mats loaded with AgNPs for wound management. The antimicrobial results of the nanofibrous mats showed superior antibacterial properties, resulting from the synergistic antibacterial effects of combining chitosan with AgNPs [89]. Zhao et al. prepared carboxymethyl-chitosan/PVA nanofibers incorporated with AgNPs with beadless surfaces and uniform diameters ranging between 295 and 343 nm. The superior antimicrobial efficacy of AgNP-loaded nanofibers against *S. aureus* revealed that these scaffolds are potential antibacterial materials for treating infected wounds [90].

Kharaghani et al. prepared chitosan/PVA nanofibers co-loaded with Ag and Cu nanoparticles for the management of infected wounds. The SEM analysis of hybrid nanofibers showed smooth nanofibrous morphology that imitates the ECM. The in vitro studies showed that the AgNP-loaded nanofibers induced superior antibacterial efficacy and good cytocompatibility compared with Cu nanoparticle-loaded nanofibers, an important feature of wound dressings for treating infected wounds [91]. Liu et al. fabricated chitosan-based nanofibers co-encapsulated with AgNPs and curcumin for wound treatment. The in vivo wound closure studies using male Kunming mice showed that the dual drug-loaded nanofibers promoted a significant decrease in skin defects, with a reduced scar [92].

The nanofibers fabricated from chitosan and other polymers significantly displayed promising mechanical properties that are similar to human skin, indicating their compatibility with skin. The SEM micrographs of nanofibers showed bead-free nanofibrous morphology that mimics the ECM of the skin, further confirming compatibility with human skin. Chitosan-based nanofibers incorporated with Ag nanoparticles also demonstrated superior antibacterial efficacy and high cell viability of skin cells. The in vitro drug release profiles demonstrated an initial burst drug release mechanism of Ag nanoparticles from chitosan nanofibers followed by a sustained release mode, suggesting that Ag nanoparticle-loaded nanofibers treat infected wounds and further prevent wounds from infection. The antibacterial studies of chitosan nanofibers also showed superior antibacterial efficacy in comparison with pristine nanofibers.

### 5.3. Hydrogels

**Figure 5:**
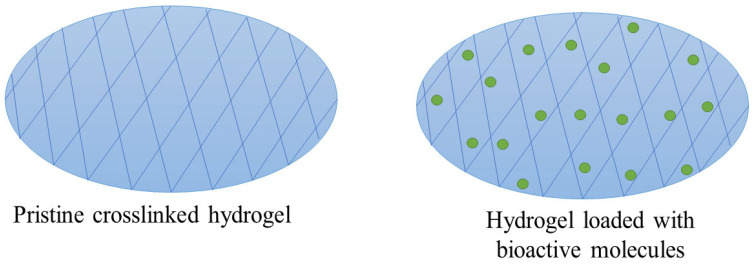
Schematic diagram showing pristine crosslinked hydrogel and drug-loaded hydrogel.

Hydrogel dressings are 3D polymeric networks comprising chemically or physically crosslinked bonds of hydrophilic biomaterials (Figure 5). The hydrophilic structures that are insoluble revealed a notable effectiveness in allowing oxygen diffusion and absorption of wound exudates to stimulate the acceleration of wound healing [93]. Significantly, hydrogels have an extremely hydrated polymeric 3D network and possess the capability to absorb several-fold water in comparison to their dry weight, thereby sustaining a high degree of moisture on the wound bed. These unique physical features can change hydrogels into different shapes and sizes [94]. The water content of polymeric hydrogels is also a vital feature that promotes wound healing and usually ranges between 90% and 95%. This provides a moist environment to promote the rate of wound healing faster than a dry environment. It has been reported that healing in a moist environment is faster than that in a dry environment because renewed skin forms in a moist environment [95]. Hydrogels can be loaded with antimicrobial agents, GFs, vitamins, and cells, as well as diverse biomolecules [96].

Jiang et al. fabricated chitosan/Konjac glucomannan hybrid hydrogels loaded with AgNPs for wound dressing applications. The drug release kinetics displayed sustained release of Ag^+^ from the hybrid hydrogels. The in vivo experiments showed that the infected wounds treated with AgNP-incorporated hydrogels healed faster than the wounds dressed with gauze and plain hydrogels [97]. Xie et al. formulated chitosan-based hydrogels incorporated with AgNPs that exhibited superior antimicrobial efficacy against *E. coli* and *S. aureus* than the pristine hydrogels, suggesting their potential to treat infected wounds [98]. The AgNP-incorporated hydrogels reported by Nešović et al. showed good antibacterial efficacy against *E. coli* and *S. aureus*, with more than 90% cell viability towards human fibroblasts (MRC-5) and L929 cells, indicating potential antimicrobial effects and non-toxicity, useful features needed in wound dressings for treating infected wounds [99].

Chu et al. reported chitosan/sericin hydrogels co-loaded with AgNPs and lupeol. The in vivo experiments employing infected full-thickness wound models in Sprague Dawley (SD) rats showed that the dual drug-loaded hydrogels significantly accelerated the wound closure rate more than hydrogels loaded with a single bioactive agent, indicating a synergistic antimicrobial effect [100]. Kumar et al. fabricated chitosan/PVA hydrogels incorporated with AgNPs. The antimicrobial effects of the hydrogels loaded with AgNPs were significant when compared to pure hydrogels [101]. Chalitangkoon et al. formulated chitosan-based hydrogels loaded with AgNPs that exhibited antimicrobial efficacy against *S. aureus* and *E. coli* as well as excellent cytocompatibility towards Vero cells [102]. Choudhary et al. reported chitosan/ε-poly-L-lysine hybrid hydrogels loaded with graphene-AgNPs for wound dressings. The excellent tensile strength and elastic modulus of the hydrogel indicate their easy handling during application. The in vivo experiments showed that the chitosan-based hydrogels incorporated with Ag nanoparticles significantly stimulated a faster rate of wound closure than the commercially available wound dressings, (cotton gauge, Tegaderm, and Fibroheal-silver) [103].

Pandian et al. reported carboxymethyl chitosan-based hydrogels loaded with AgNPs that induced superior anti-biofilm efficacy against *S. aureus*, *P. aeruginosa*, and *E. coli*, revealing their potential application as effective antibacterial wound dressings [104]. Zhou et al. fabricated chitosan/gelatin hybrid hydrogels incorporated with AgNPs for antibacterial wound dressing. The biological studies showed that AgNP-loaded hydrogels significantly accelerated the wound-healing process and promoted better antibacterial efficacy against *E. coli* and *S. aureus* than pristine hydrogels [105]. The in vitro antimicrobial studies of green synthesized AgNPs, loaded into chitosan/PVA hydrogels prepared by Aldakheel et al. using the agar diffusion method, demonstrated outstanding antimicrobial efficacy against *E. coli* and *S. aureus* [106]. Chitosan/PVA hydrogels containing AgNPs reported by Suflet et al. showed high inhibitory activity against *K. pneumonia* and *S. aureus*, indicating that these hydrogels are effective antibacterial wound dressings [107].

Bharathi et al. reported AgNP-loaded chitosan hydrogels. The AgNPs were synthesized using *Saussurea lappa* root extract. The hydrogel promoted accelerated wound healing of excisional wounds infected with *P. aeruginosa* compared with plain hydrogels in Wistar albino rats [108]. Ferfera-Harrar et al. explored chitosan/polyacrylamide hydrogels loaded with AgNPs synthesized using *Curcuma longa* extract in wound treatment. The SEM images of the hydrogels showed smooth and porous morphology, useful for the diffusion of nutrients, water, and oxygen during wound healing. Moreover, the in vitro antibacterial studies showed that the dual drug-incorporated hydrogels possessed better antibacterial activity against *E. coli* and *S. aureus* than the plain hydrogels [109]. Khampieng et al. developed chitosan/alginate/PVP hybrid hydrogels containing AgNPs for wound dressing. The swelling analysis of the hybrid hydrogels was more than 1500% after 24 h, indicating an excellent capacity to absorb high volumes of exudates and also maintain a moist environment. The antibacterial efficacy of the hydrogels was enhanced against various bacteria strains (i.e., *P. aeruginosa*, *S. aureus*, *E. coli*, and MRSA) and influenced by the amount of AgNPs loaded into the hydrogels [110].

Lee et al. fabricated chitosan-based hydrogels co-loaded with AgNPs and epidermal GFs for diabetic wound treatment. The in vivo analysis of the dual drug-incorporated hydrogels using diabetic rats showed improved wound repair effects than the commercially available dressings, (i.e., gauze and HeraDerm). The hydrogels showed significant antibacterial effects against *S. aureus* and *S. epidermidis*, demonstrating their potential application for the treatment of infected diabetic injuries [111]. The chitosan/PEG hydrogels reported by Masood et al. showed a sustained drug release of AgNPs with enhanced wound healing effects on diabetic rabbits and superior broad-spectrum antibacterial activity against *P. aeruginosa*, *E. coli*, *S. aureus*, *B. pumilus*, and *B. subtilis* [112].

The excellent mechanical properties of chitosan-based hydrogels make their handling easy when applied on wounds. The in vitro cytotoxicity studies showed non-toxicity and excellent cytocompatibility when Ag nanoparticle-incorporated chitosan hydrogels were incubated with various skin cells. The in vivo wound healing analysis further demonstrated that chitosan hydrogels loaded with Ag nanoparticles significantly accelerate the rate of wound closure of various wound models compared with plain hydrogels. Moreover, the in vitro antibacterial experiments of chitosan-based hydrogels loaded with Ag nanoparticles indicated excellent antimicrobial effects against several bacterial strains. These biological outcomes revealed that chitosan-based hydrogels incorporated with Ag nanoparticles are potential wound dressings for the treatment of injuries, including infected wounds.

### 5.4. Cryogels

**Figure 6:**
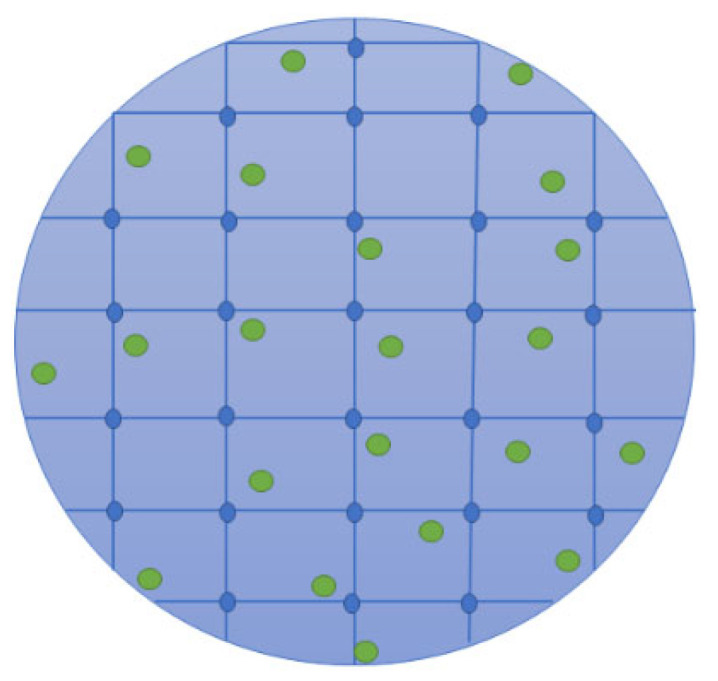
A schematic diagram of a cryogel loaded with bioactive agents.

Cryogels (Figure 6) are gels that are dried into highly absorbent and porous scaffolds with a high surface area. Several methods can be used to prepare cryogels, such as freeze drying, ambient pressure drying, supercritical drying, vacuum drying, and microwave drying [113]. Cryogels have been explored as drug delivery systems and wound dressings, used in supercapacitors, multifunctional sensors, insulators, etc. [114]. Their absorption capacity is a significant parameter for many biomedical applications and is associated with their internal surface area, crosslinking density, and charged functional groups [115]. The various crucial properties of cryogels have promoted their application by biomedical researchers, including their high specific surface area, porous nature that promotes high drug load capacity, amorphous state that helps in stabilizing drugs towards recrystallization, moderate drug release mechanism, etc. [116,117]. Several researchers have investigated chitosan-based cryogels containing AgNPs for the treatment of infected wounds.

Xu et al. designed chitosan/gelatin cryogels co-loaded with AgNPs and tannic acid to treat bacterial-infected wounds [118]. The hemostasis studies of chitosan-based cryogels using a liver bleeding rat model showed an accelerated hemostasis compared with gauze because of their porous structure, revealing their potential application for managing bleeding injuries. The in vivo wound repair studies utilizing a murine full-thickness wound model infected by *S. aureus* showed that the dual drug-loaded cryogels effectively eradicated bacteria, resulting in a higher wound closure rate than the pristine chitosan cryogels. The in vitro studies further confirmed the effective antimicrobial activity of cryogels containing AgNPs and tannic acid, with more than 99% bactericidal effects against *E. coli* and *S. aureus* [118]. The chitosan/PEG cryogels containing AgNPs reported by Zou et al. showed superior antimicrobial activity against *E. coli*, revealing them as promising materials for the management of infected chronic wounds [119].

Demir et al. reported chitosan-based cryogels incorporated with AgNPs for the treatment of infected injuries [120]. The SEM pictures of AgNP-incorporated cryogels exhibited an interconnected porous structure, with pore sizes that ranged from 5 to 30 μm. The structure was effective in supporting cell adhesion, movement, and proliferation during wound healing and skin regeneration. The in vitro antioxidant analysis of cryogels containing 100 mg/L of AgNPs employing the DPPH process exhibited more than 85% scavenging efficacy, revealing their potential application as free radical scavengers. The antimicrobial studies of AgNP-loaded cryogels showed good broad-spectrum bactericidal effects that were nanoparticle concentration-dependent, indicating their effectiveness in the management of wounds infected by various bacteria strains [120]. Mohammed et al. fabricated chitosan-based cryogels containing AgNPs synthesized from *Rosmarinus officinalis* leaf extract. The AgNP-loaded cryogels exhibited excellent antibacterial efficacy that was concentration-dependent against several bacteria strains [121].

The cryogels loaded with Ag nanoparticles displayed accelerated hemostasis when compared to other biomaterials (e.g., gauze), indicating the effectiveness for the management of bleeding wounds. Some reports showed that chitosan-based cryogels significantly eradicated bacteria from skin defects in vivo, with an accelerated rate of wound healing mechanism. Furthermore, the in vitro antibacterial studies of chitosan-based hydrogels demonstrated excellent antibacterial activities against several bacteria strains that are common in clinically infected wounds.

### 5.5. Films and Membranes

**Figure 7:**
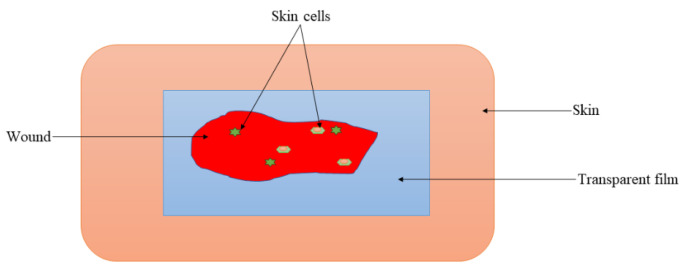
Transparent film for wound dressing.

Film dressings (Figure 7) are polymeric scaffolds that are typically prepared from transparent and adherent PUs that allow gaseous exchange between the wound bed and the environment [122]. Films are very useful for the autolytic elimination of debris from the wound. Some fascinating features are displayed by film wound dressings, such as high elasticity and flexibility, that make them change to any shape without the need for additional taping [123]. Due to the transparency of the films, the wound-healing process can be monitored. These wound dressings are suitable for superficial, epithelizing, shallow injuries with low wound exudates [124]. Zhao et al. prepared chitosan/cellulose hybrid films (Figure 7) that demonstrated superior antibacterial efficacy compared with pristine hybrid films against Gram-positive and Gram-negative bacteria strains, with good cytocompatibility towards NIH3T3 fibroblasts, suggesting effective antibacterial activity and non-toxicity [125].

Choudhary et al. fabricated chitosan/graphene/ε-poly-L-lysine films loaded with AgNPs for antibacterial wound care. The hybrid films displayed an elastic modulus and tensile strength of 361.56 ± 17.89 and 78.9 ± 3.99 MPa, respectively, indicating excellent mechanical performance suitable for wound dressing. The in vitro antimicrobial studies showed that the films loaded with AgNPs displayed excellent antimicrobial effects against *E. coli* and *S. aureus*, with a killing efficiency of approximately 99.99% when compared to plain chitosan films, which showed only 90% killing efficiency [103]. Tang et al. formulated chitosan/sodium cellulose sulfate composite films containing AgNPs that showed a water-vapor transmission rate (WVTR) of 1053.8 ± 19.1 g·m^−2^·d^−1^, porosity that ranged from 74.23 ± 2.84% to 93.57 ± 1.33%, and a swelling capacity of 1670.7 ± 37.5%, demonstrating good capability to absorb a large amount of exudate and provide suitable moisture for accelerated wound healing without scar formation. The composite films were effective against *E. coli* and *S. aureus*, making the chitosan films potential wound dressings for the treatment of infected wounds [126]. The chitosan films containing AgNPs reported by Vimala et al. noticeably resulted in superior growth inhibition of *E. coli*, *K. pneumoniae*, and *Bacillus* compared with plain films [127].

Bajpai et al. fabricated chitosan-based films incorporated with green synthesized AgNPs using curcumin for wound dressing applications. The skin irritation analysis using a shaved skin rat model showed that the dual drug-loaded films did not induce skin irritation, while the films loaded with only Ag nanoparticles demonstrated some signs of skin irritation. Moreover, the in vivo experiments showed that the dual drug-loaded films significantly accelerated wound reduction in comparison to films loaded with AgNPs alone [128]. Nguyen et al. prepared chitosan/pectin composite films impregnated with AgNPs synthesized using *Piper betle* leaf extract for antibacterial wound dressing applications. These films demonstrated excellent antimicrobial effects against *P. aeruginosa*, *S. aureus*, *K. pneumonia*, and *B. cereus*. Moreover, the in vivo outcomes of the films containing AgNPs exhibited complete wound closure after 15 days, suggesting their potential to accelerate the rate of wound healing [129].

Arockianathan et al. formulated chitosan/sago starch hybrid films co-incorporated with AgNPs and gentamicin for wound treatment. The wound repair analysis showed that the dual drug-incorporated films and chitosan films containing AgNPs significantly accelerated wound closure rate more than sterile cotton gauze. The antimicrobial effects of the drug-loaded films accelerated the rate of wound healing [130]. Shah et al. prepared chitosan/sericin films co-incorporated with AgNPs and moxifloxacin for wound healing. The in vitro antimicrobial evaluation of the dual drug-loaded films showed good bactericidal activity against *S. epidermidis*, *S. aureus*, MRSA, *A. baumannii*, and *P. aeruginosa*. The in vivo wound closure studies utilizing burn-injured rat models revealed that the dual drug-loaded films significantly induced more rapid wound closure than the plain chitosan films [131]. Hasibuan et al. synthesized chitosan/cellulose composite films incorporated with AgNPs. The in vitro experiments revealed that the films containing nanoparticles induced the strongest inhibition zone when cultured with *B. subtilis* and *P. aeruginosa*, revealing excellent antibacterial activity suitable for bacterial-infected wound care [132].

Chitosan films containing AgNPs fabricated by Ambrogi et al. displayed suitable WVTR for wound healing and demonstrated good antibacterial efficacy against *P. aeruginosa*, *S. aureus*, and *S. epidermidis* [133]. Cadinoiu et al. formulated chitosan/PVA films containing AgNPs and ibuprofen that exhibited superior antibacterial efficacy against *S. aureus*, with good cytocompatibility towards HDFa cell lines and an 89% cell viability, suggesting good antibacterial activity and non-toxicity [134]. Chitosan-based films containing AgNPs developed by Thomas et al. exhibited superior antibacterial activity against *Bacillus* and *E. coli* [135]. Dong and Li fabricated chitosan/cellulose nanocrystal films loaded with Ag nanoparticles for antibacterial wound healing. The maximum swelling ratios of the films ranged between 356% and 410%, depending on the content of AgNPs and cellulose nanocrystals, revealing good water barrier features that result in high absorption of the exudate and a moist healing environment. Moreover, the film dressings were active against Gram-positive and Gram-negative bacteria strains, revealing their potential application as antibacterial wound dressings [136].

Membranes are wound dressing materials that possess similar features to films. However, membranes’ ability to absorb excess exudate, keep biological fluids under pressure, maintain suitable moisture for rapid wound repair mechanism, possess effective cleaning activity, not need frequent dressing changes, and reduce the disruption of the wound bed make them interesting wound dressings for infected wounds [21,137]. Furthermore, membranes display excellent mechanical features, such as stretchability, softness, and flexibility [137]. Nhi et al. synthesized chitosan/PCL membranes loaded with AgNPs for antibacterial wound dressing applications. The mechanical properties of Ag nanoparticle-loaded membranes showed a tensile stress of 7.5 MPa and a strain of 90%. The chitosan-based hybrid membranes displayed good antibacterial efficacy against *P. aeruginosa*, *E. coli*, and *S. aureus*, with high cell proliferation when incubated with L-929 cells, demonstrating a non-toxic effect [138].

Tang et al. fabricated chitosan/PCL hybrid membranes loaded with AgNPs. The WVTR values of membranes ranged between 2652.03 ± 185.51 and 3345.50 ± 122.34 g/m^2^/24 h, demonstrating their capability to maintain moisture during the wound-healing process. The release of nanoparticles from the dressings was characterized by an initial rapid release followed by a sustained drug release. The membranes containing AgNPs significantly resulted in larger inhibition zones when compared to plain membranes, revealing an excellent antibacterial efficacy of Ag nanoparticles in the membranes [139]. El-Aassar et al. fabricated chitosan/gelatin/PVP hybrid membranes encapsulated with AgNPs green synthesized utilizing *Citrullus colocynthis* plant extract. The membranes containing nanoparticles promoted stronger antibacterial activity than the pristine membranes against *S. typhi*, *E. coli*, *B. subtilis*, and *S. aureus* in a dose-dependent manner, suggesting that they are effective antibacterial wound dressing scaffolds [140].

The chitosan-based films and membranes loaded with Ag nanoparticles exhibited auspicious therapeutic outcomes, making them suitable materials for the treatment of infected wounds. These materials displayed excellent mechanical performance, a crucial feature in the management of wounds. The in vitro experiments of chitosan-based films and membranes incorporated with Ag nanoparticles showed superior antibacterial activity in comparison to plain scaffolds with non-toxicity and good cytocompatibility towards skin cells. Moreover, these films and membranes displayed desirable porosity, WVTR, and swelling capacity that can offer appropriate moisture for the acceleration of wound healing.

### 5.6. Foams and Wafers

Foams are porous solid wound dressing scaffolds that are composed of hydrophilic and hydrophobic material with bio-adhesive boundaries [141]. The hydrophobic layer protects the wound from liquids and permits water-vapor diffusion and gaseous exchange. Foams can be sterilized and used in injuries without resulting in pain to the patient if their parameters, such as mechanical features, are tailored appropriately [139]. The advantages of foams include their capacity to prevent maceration on the injury, enhance gaseous exchange, offer appropriate moisture for wound beds, and absorb excess wound exudate, making them suitable for treating diabetic ulcers, burns, etc. [142]. However, foams are inappropriate for dry wounds or wounds with low exudates [143]. Guibal et al. prepared chitosan-based foams loaded with AgNPs. The in vitro studies revealed that an increase in the content of AgNPs from 0.2 to 1.82 mg per disk significantly enhanced the antibacterial effects of the foams against various bacterial strains (*S. aureus*, *S. hominis*, and *P. aeruginosa*) [144].

Biswas et al. prepared chitosan/PVA foams loaded with AgNPs with a highly porous morphology to support cell migration and proliferation during wound healing mechanisms. The hybrid foams containing AgNPs displayed good antibacterial efficacy against *E. coli*, *S. aureus*, and MRSA that was concentration-dependent, indicating their effectiveness as antimicrobial wound dressing materials [145]. Permyakova et al. reported chitosan/curdlan hybrid foams incorporated with AgNPs for wound dressing applications. The in vivo evaluation employing a full-thickness skin wound model in diabetic mice showed that the foams containing AgNPs significantly accelerated the rate of wound contraction compared with plain hybrid foams [146].

On the other hand, wafers are also porous dressings prepared by a lyophilization method. They have been utilized for treating infected wounds. They absorb exudates and turn into a gel, providing moisture for accelerated wound healing [147]. The advantages of wafer dressings are their capability to act as topical drug delivery systems, their mucoadhesive nature, prolonged retention on the injury, and their capacity to be incorporated with both insoluble and soluble bioactive molecules. The preparation process of wafers is vital because poor formulation processes from poor ratios of materials can result in sticky, rigid, and non-porous wafers unsuitable for wound dressing applications [143]. Jaiswal et al. prepared chitosan-based wafers loaded with AgNPs for wound healing applications. These wafers significantly enhanced the mechanical properties of PVA hydrogels by exhibiting maximum elongation, tensile strength, and elastic modulus of 244 ± 7%, 0.279 ± 0.06 MPa, and 0.62 ± 0.13 PSI, respectively. The PVA hydrogels loaded with chitosan wafers containing AgNPs showed good antibacterial activity in vitro and significantly stimulated a higher rate of wound contraction in vivo than the cotton gauze [148].

The chitosan foams showed high porosity that can significantly support cell migration and proliferation during the process of wound healing. The chitosan-based wafers displayed good mechanical properties that can be beneficial during wound care. Both chitosan-based foams and wafers demonstrated superior antibacterial activity against various bacterial strains and significantly induced a higher rate of wound healing when compared to plain chitosan materials and other currently used wound dressings (e.g., cotton gauze). A summary of chitosan-based wound dressings loaded with AgNPs [65,66,67,68,69,70,71,72,73,74,75,76,77,78,79,80,81,82,83,84,85,86,87,88,89,90,91,92,93,94,95,96,97,98,99,100,101,102,103,104,105,106,107,108,109,110,111,112,113,114,115,116,117,118,119,120,121,122,123,124,125,126,127,128,129,130,131,132,133,134,135,136,137,138,139,140,141,142,143,144,145,146,147,148] is shown in Table 2.

## 6. Clinical Trials of Chitosan-Based Wound Dressings

There are few reports on the clinical trials of different chitosan-based wound dressings. Mo et al. evaluated the efficacy of acrylated chitosan-based fibers developed by Foshan United Medical Technologies Ltd (Guangdong, China). in a randomized clinical study involving 90 patients in China [149]. The control used was a sterile Vaseline gauze. The chitosan-based wound dressing exhibited higher absorbent capability than the Vaseline gauze dressing and reduced the wound area and depth by 65.97 ± 4.48% compared with the control group, 39.95 ± 4.48%, after 4 weeks of treatment. The healing rate of the chitosan wound dressings was 43% higher than the control group, with a healing rate of 11.7% in chronic wounds [149]. Abdollahimajd et al. compared the clinical efficacy and safety of chitosan and nanosilver (ActicoatTM) dressings in refractory diabetic wounds. The study involved 25 Patients with chronic diabetic wounds. Chitosan dressing was used on 13 patients and nanosilver on 12 patients. The chitosan wound dressing was as safe and effective as the nano-silver (ActicoatTM) dressing [150]. Halim et al. reported findings from clinical trials on 244 patients in which the wound-healing effect of chitosan-based films and hydrocolloids were compared. The group treated with chitosan films exhibited reduced odour and exudate than the hydrocolloid group. However, there were no significant differences between the chitosan film and hydrocolloid groups in terms of ease of removal, adherence, itchiness, tenderness, pain, erythema, and wound drainage, indicating that chitosan-based films can be used as an option in the treatment of abrasion and superficial injuries [151].

Hu et al. evaluated chitosan wound dressings combined with wet dressings in patients with deep second-degree burns. The clinical study was carried out on 80 patients between 2019 and 2021. The wound healing time of the chitosan wound dressing combined with the wet dressing group was shorter (19.53 ± 2.74 days) when compared to that of the wet dressing alone group (24.78 ± 4.86 days). The scar score of the chitosan wound dressing combined with the wet dressing was lower than that of the wet dressing (reference group) [152]. A few reports revealed the efficacy of chitosan-based wound dressings in wound healing. However, more studies are needed to compare the amount of scarring, regeneration time, and their antibacterial effects. The clinical outcomes of chitosan-based wound dressings revealed an accelerated rate of wound healing, and the capability to absorb wound exudates and reduce the wound area when compared to other classes of wound dressings used as controls.

Most of the currently used wound dressing materials suffer from several limitations. Hydrogels prepared from silk fibroin suffer from limitations in wound healing, such as extended time of gelation, organic solvent or high temperature-assisted treatment, and the absence of self-healing properties [153]. Gauze wound dressings need frequent changing of dressing to protect healthy tissues from maceration. These wound dressings become moistened and adherent to the wound bed when used in high-exuding wounds, resulting in pain during wound dressing removal. Semi-permeable film dressings that are originally derived from nylon suffer from poor absorption, making them inappropriate for high-exuding wounds, and they can also cause wound maceration. Hydrocolloids are not suitable for high-exuding wounds or neuropathic ulcers, and they are commonly employed as secondary wound dressings. The limitation of alginate dressings is that they also require secondary dressings because of their ability to cause wound dehydration, which delays wound healing. Moreover, composite wound dressings possess less flexibility and are expensive [141]. Several chitosan-based wound dressing products are commercially available for the treatment of wounds. Table 3 below depicts some of those wound dressing products [47,48,154,155,156,157,158,159,160,161,162,163]. Although several chitosan-based wound dressing products are currently available on the market, most of them are only suitable for high-exudate and bleeding wounds.

## 7. Conclusions

Chitosan-based wound dressing scaffolds are useful for the treatment of infected injuries due to the antimicrobial effects of chitosan. However, preclinical reports have revealed that pristine chitosan wound dressings possessed poor antimicrobial properties against several bacterial strains that are common in wounds. A series of in vivo and in vitro experiments demonstrated that incorporating AgNPs into the chitosan scaffolds resulted in promising infection control. The chitosan dressings containing AgNPs demonstrated an excellent antibacterial efficacy of more than 90% compared with plain chitosan wound dressings against various bacterial strains (i.e., *P. aeruginosa*, *S. aureus*, *E. coli*, etc.). Scaffolds such as hydrogels, films, and nanofibers displayed high antimicrobial activity when compared to other chitosan-based scaffolds. However, the fabrication of wound dressings incorporated with silver nanoparticles has not been fully explored, especially for wounds requiring immediate treatment. However, topical wound dressings, such as topical gels, are more appropriate for treating wounds requiring immediate treatment. The antibacterial activity of AgNPs is due to their capability to destroy bacterial membranes, causing intracellular death, while chitosan, on the other hand, hinders bacterial growth due to its cationic nature. The antimicrobial effects of chitosan dressings were enhanced as the content of Ag nanoparticles increased, demonstrating a dose-dependent manner. However, the amount of metallic nanoparticles must be thoroughly monitored because high concentrations can result in significant toxicity to skin cells. The reported AgNP-loaded chitosan dressings displayed good cell viability that ranged between 89% and 93.5%, revealing good biocompatibility and reduced toxicity. These outcomes revealed that chitosan wound dressings loaded with Ag nanoparticles are potential wound dressings that need further studies. Further biological studies and a thorough understanding of the mode of healing of these wound dressings will result in new dressings that can overcome the problem of delayed wound healing that is common in infected wounds.

## 8. Future Perspective

The chitosan-based wound dressings loaded with AgNPs demonstrated superior antibacterial studies with good biocompatibility and low toxicity, making them potential candidates for the treatment of infected wounds. There is a need for researchers to explore the loading of green synthesized AgNPs into chitosan dressings to further reduce drug toxicity. The co-loading of AgNPs with other bioactive molecules into the chitosan scaffolds can further enhance the antimicrobial effects. Other bioactive agents that should be investigated for drug co-loading with AgNPs into the chitosan wound dressings are essential oils. The possible synergistic antimicrobial effects of chitosan in combination with AgNPs are unclear, indicating a knowledge gap that must be studied in these biomaterials for antibacterial wound dressing applications. Moreover, the fabrication of AgNP-incorporated chitosan dressings for the management of infected wounds employing new techniques such as 3D printing is a future area of research that should be explored. Most of the reported chitosan-based wound dressings loaded with AgNPs are not designed for infected wounds requiring immediate infection treatment. There is a pressing need for the fabrication of topical wound dressings incorporated with silver nanoparticles for wounds requiring immediate treatment. These future developments can solve several clinical challenges that are currently posed by chronic infected wounds. They can overcome issues of biofilm formation, chronicity, and harsh conditions in the lesion microenvironment, which normally limit the required amounts of bioactive antimicrobials at the infected wound site. Moreover, the problem of cytotoxicity that is frequently caused by many of the presently used therapies (e.g., antibiotics) for the treatment of infected wounds can also be resolved.

## Figures and Tables

**Table 1:** Summary of the classes of wound dressings.

Types of Wound Dressing	Description	Advantages	Limitations	References
Traditional dressings (e.g., gauze, wool dressing, bandages, plaster, and gauze)	Traditional wound dressings are dry and utilized as secondary dressings for protecting the injury from contamination. These dressings have been widely utilized since the 19th century because of their simple manufacturing, low cost, and easy application. Traditional dressings are normally employed for wounds with mild exudate.	Protect wounds from contamination, cushion the injury, absorb wound fluids, and terminate bleeding.	Leakage of exudate that promotes bacterial invasion of the wound.	[33,34]
Skin substitutes (e.g., TransCyte, OrCel, and Apligraf)	There are two types of skin substitutes: cellular (which imitates the skin layer made up of fibroblasts and Keratinocytes) and acellular matrix (which contains only the dermal elements with fibroblasts). The main mechanism of skin substitutes is to release and activate growth factors by which epithelialization is accomplished. These dressings are appropriate for venous leg ulcers and diabetic foot ulcers.	Effective in skin regeneration.	Transmission of d34iseases and wound infections, are expensive, can be rejected by the immune system of the host, and possess inadequate shelf life.	[35]
Dermal grafts (e.g., Acellular xenografts, allografts, and autografts)	Dermal grafts are the important dressings in dermatology and plastic surgery. These wound dressings are normally not considered for the management of complex lesions. They are used in various clinical situations, including traumatic wounds, burn reconstruction, defects after oncologic resection, etc.	Effective for the treatment of numerous wounds, such as oncologic resection, traumatic lesions, burns, etc.	Inability to treat complex wounds.	[36]
Interactive dressings (e.g., acellular xenografts, allografts, and autografts)	Interactive dressings are primarily used to protect wounds from bacterial infections. They are frequently fabricated from synthetic polymers. These wound dressings can be used for the treatment of venous stasis ulcers, pressure ulcers, arterial ulcers, and burns.	Offer moisture and enhance re-epithelialization and granulation process.	Poor antimicrobial effects.	[37]
Bioactive dressings (e.g., sponges, hydrocolloids, wafers, hydrogels, nanofibers, foams, and films)	Bioactive dressings are wound dressings that are mainly used for drug delivery of various bioactive agents (e.g., antibacterial molecules, growth factors, vitamins, etc.). They induce tissue repair, especially when the natural healing processes of the body are compromised. Bioactive dressings are effective for chronic injuries such as pressure, diabetes, and venous ulcers.	Good biocompatibility and biodegradability and are drug-delivery systems.	No obvious shortcomings.	[38,39]

**Table 2:** Summary of chitosan-based wound dressings loaded with AgNPs.

Types of Wound Dressing	Polymers Combined Chitosan	Preclinical Studies (In Vivo or In Vitro)	Significant Therapeutic Outcomes	References
Sponges	Gelatin	In vitro cytotoxicity and antibacterial studies	High cell viability towards skin cells and good antibacterial activity	[65]
Sponges	Alginate	In vitro antimicrobial analysis	Potential antibacterial efficacy against *P. aeruginosa* and *S. aureus*	[66]
Sponges	_	In vitro antibacterial experiments	High inhibition efficiency against *S. aureus* and *P. aeruginosa*	[67]
Sponges	Silk fibroin	In vitro antibacterial and in vivo wound healing studies	Superior antibacterial activity and accelerated wound-healing process of 99.38% on the 14th.	[68]
Sponges	_	In vitro antimicrobial and in vivo wound healing studies	Superior antibacterial effects against *E. coli*, *P. aeruginosa*, *S. aureus*, and MRSA with excellent healing activity compared to ZnO ointment gauze.	[69]
Sponges	_	In vitro antimicrobial and cell viability analysis	Antibacterial activity against *E. coli* and *S. aureus* with non-toxicity towards MC3T3 cells	[70]
Sponges	_	In vitro antimicrobial and cell proliferation analysis	Effective antibacterial outcomes and promoted cell migration and proliferation	[71]
Sponges	Hyaluronic acid	In vitro antimicrobial and in vivo wound healing studies	High inhibition of bacterial growth and faster wound contraction.	[72]
Sponges	_	In vitro antibacterial studies	Excellent antibacterial studies against *E. coli*, *P. aeruginosa*, and *S. aureus*.	[73]
Sponges	*Bletilla striata* polysaccharide	In vitro antimicrobial and in vivo wound healing studies	Potent antibacterial against *E. coli*, *P. aeruginosa*, and *S. aureus* with faster rate and wound healing.	[74]
Sponges	Alginate	In vitro antimicrobial studies	Greater bacterial inhibition than gentamicin	[75]
Nanofibers	PEO	In vitro antimicrobial studies	Good antibacterial efficacy against *P. aeruginosa* and *S. aureus*	[79]
Nanofibers	Silk fibroin	In vitro wound healing and antibacterial studies	Good wound healing effects and stronger bacterial inhibitory effects	[80]
Nanofibers	PVA	In vitro drug release studies	Sustained drug release profile	[81]
Nanofibers	_	In vitro drug release studies	Effective antibacterial effects against MRSA and *P. aeruginosa*	[82]
Nanofibers	PEO	In vitro antimicrobial and cell viability analysis	Superior antibacterial efficacy against *S. aureus* and *E. coli* with high cell viability of 93.5%	[83]
Nanofibers	PLA	In vitro antimicrobial and cytotoxicity analysis	Good antibacterial efficacy against *S. aureus* and *E. coli* with excellent cytocompatibility	[84]
Nanofiber	PVA and PCL	In vitro drug release and antimicrobial studies	Controlled drug release and superior antibacterial efficacy against *E. coli* and *S. aureus*	[85]
Nanofibers	PEO	In vitro antimicrobial analysis	Excellent antibacterial effects against *S. aureus* and *E. coli*	[86]
Nanofibers	PEO	In vitro antibacterial and cytotoxicity experiments	Good antibacterial efficacy and excellent cytocompatibility	[87]
Nanofibers	PEO	In vitro drug release and antimicrobial studies	Sustained drug release and excellent antibacterial activity	[88]
Nanofibers	PVA	In vitro antimicrobial studies	Superior antibacterial properties	[89]
Nanofibers	PVA	In vitro antimicrobial studies	Superior antimicrobial efficacy against *S. aureus*	[90]
Nanofibers	PVA	In vitro antibacterial studies	Superior antibacterial efficacy	[91]
Nanofibers	_	In vivo wound closure studies	Improved the wound healing rates	[92]
Hydrogels	Konjac glucomannan	In vitro drug release and in vivo antimicrobial studies	Sustained drug release mechanism and accelerated wound healing rate.	[97]
Hydrogels	_	In vitro antibacterial studies	Superior antimicrobial activity against *E. coli* and *S. aureus*	[98]
Hydrogels	_	In vitro antibacterial and cytotoxicity experiments	Good antibacterial efficacy against *S. aureus* and *E. coli* and high cell viability towards skin cells	[99]
Hydrogels	Sericin	In vivo studies using infected full-thickness wound models	Accelerated the wound closure rate	[100]
Hydrogels	PVA	In vitro antibacterial studies	Superior antimicrobial activity against *E. coli* and *S. aureus*	[101]
Hydrogels	_	In vitro antibacterial and cytotoxicity experiments	Antibacterial efficacy against *E. coli* and *S. aureus*	[102]
Hydrogels	ε-poly-L-lysine	In vivo wound healing studies	Accelerated rate of wound healing	[103]
Hydrogels	_	In vitro antibacterial studies	superior anti-biofilm efficacy against *P. aeruginosa*, *E. coli*, and *S. aureus*	[104]
Hydrogels	Gelatin	In vitro wound healing and antibacterial studies	Accelerated wound-healing process with superior antibacterial efficacy against *E. coli* and *S. aureus*	[105]
Hydrogels	PVA	In vitro antibacterial studies	Excellent antibacterial efficacy against *S. aureus* and *E. coli*	[106]
Hydrogels	PVA	In vitro antimicrobial studies	High inhibitory effect against *K. pneumonia* and *S. aureus*	[107]
Hydrogels	_	In vivo antibacterial wound healing studies	Accelerated wound healing of excisional wounds infected with *P. aeruginosa*	[108]
Hydrogels	Polyacrylamide	In vitro antimicrobial studies	Superior antibacterial efficacy against *S. aureus* and *E. coli*	[109]
Hydrogels	Alginate and PVP	In vitro antimicrobial studies	Superior antibacterial efficacy against *P. aeruginosa*, *E. coli*, *S. aureus*, and MRSA	[110]
Hydrogels	_	In vivo wound healing and in vitro antimicrobial studies	Enhanced diabetic wound repair effects and effective antibacterial effects against *S. aureus* and *S. epidermidis*	[111]
Hydrogels	PEG	In vivo wound healing and in vitro antimicrobial studies	Sustained drug release, enhanced wound healing effects, and superior broad-spectrum antibacterial efficacy.	[112]
Cryogels	Gelatin	In vivo wound healing and in vitro antimicrobial studies	Accelerated wound closure.	[118]
Cryogels	PEG	In vitro antibacterial studies	Excellent antibacterial efficacy against *E. coli*	[119]
Cryogels	_	In vitro antioxidant and antibacterial studies	Good antioxidant and antibacterial efficacy	[120]
Cryogels	_	In vitro antibacterial studies	Excellent antibacterial efficacy	[121]
Films	Cellulose	In vitro antibacterial and cytotoxicity studies	Superior antibacterial efficacy and excellent cytocompatibility	[125]
Films	Graphene and ε-poly-L-lysine	In vitro antibacterial studies	Excellent antibacterial effects	[103]
Films	Sodium cellulose sulfate	In vitro antibacterial studies	Antibacterial activity against *E. coli* and *S. aureus,*	[126]
Films	_	In vitro antimicrobial studies	Superior growth inhibition of *E. coli*, *K. pneumoniae*, and *Bacillus*	[127]
Films	_	In vivo wound closure studies	Accelerated wound reduction	[128]
Films	Pectin	In vivo wound healing and in vitro antimicrobial studies	Excellent antimicrobial effects and accelerated wound healing	[129]
Films	Sago starch	In vivo wound healing	Accelerated wound closure rate	[130]
Films	Sericin	In vivo wound healing and in vitro antimicrobial studies	Good bactericidal efficacy and rapid wound closure	[131]
Films	Cellulose	In vitro antimicrobial studies	Excellent antibacterial efficacy	[132]
Films	_	In vitro antimicrobial studies	Good antibacterial efficacy	[133]
Films	PVA	In vitro antibacterial and cytotoxicity studies	Superior antibacterial efficacy against *S. aureus* and cytocompatibility towards HDFa cell lines	[134]
Films	_	In vitro antimicrobial studies	Superior antibacterial activity	[135]
Films	Cellulose	In vitro antimicrobial studies	Good antibacterial efficacy	[136]
Membranes	PCL	In vitro antibacterial and cytotoxicity studies	Good antibacterial efficacy against *P. aeruginosa*, *E. coli*, and *S. aureus* with high cell proliferation.	[138]
Membranes	PCL	In vitro antimicrobial studies	Excellent antibacterial efficacy.	[139]
Membranes	Gelatin and PVP	In vitro antimicrobial studies	Superior antibacterial activity.	[140]
Foams	_	In vitro antimicrobial studies	Enhanced antibacterial effects	[144]
Foams	PVA	In vitro antimicrobial studies	Good antibacterial efficacy against *E. coli, S. aureus*, and MRSA	[145]
Foams	Curdlan	In vivo wound healing studies	Accelerated rate of wound contraction	[146]
Wafers	_	In vivo wound healing and in vitro antimicrobial studies	Good antibacterial activity and a higher rate of wound contraction	[148]

**Table 3:** Commercially available wound dressing products.

Products	Wound Dressing Type	Therapeutic Outcomes	References
Axiostat^®^	Sponge	Acts as a hemostatic material to terminate severe to moderate bleeding caused by abrasions, cuts, punctures, lacerations, and arterial or venous bleeding.	[154]
Celox™	Granules	Works by stimulating clot development via adsorption and dehydration and inducing the bonding of red blood cells.	[155]
ChitoSAM™ 100	Non-woven chitosan dressing	Manufactured to rapidly terminate chronic bleeding, and its ease of use is very effective.	[156]
HemCon^®^ Strip PRO	Bandage	Bio-adhesive nature results in sealing the lesion and controls bleeding.	[157]
PosiSep^®^	Hemostatic sponge	Minimizes bleeding and oedema post-surgery.	[158]
Chitoflex^®^ HemCon	Gel	Good biocompatibility and antibacterial efficacy.	[48]
Tegasorb^®^ 3M	Hydrogel	Swells when it is absorbing exudate and forms a soft gel. Suitable for chronic wounds.	[47]
Chitopack C^®^ Eisai	Gel	Completely repairs disrupted body tissues and induced skin regeneration.	[159]
Chitoseal^®^ Abbott	Gel	Possesses good hemostatic functions and biocompatibility. Appropriate for bleeding lesions.	[159]
Chitoderm^®^ plus	Gel	Good absorbent characteristics	[160]
KytoCel	Fiber	An extremely absorbent wound dressing appropriate for the treatment of high-exuding wounds.	[161]
ExcelArrest^®^ XT	Patch	Accelerates the clotting process to control bleeding from the skin.	[162]
ChitoRhino	Gel	Excellent hemostasis efficacy and potential for wound repair process after endoscopic sinus surgery	[158]
ChitoHeal	Gel	Accelerates the process of wound healing; biocompatible; reduces scar formation; effective for diabetic foot ulcers, burns, scratches, and cuts.	[163]
